# Collaborative approach to Optimise MEdication use for Older people in Nursing homes (COME-ON): study protocol of a cluster controlled trial

**DOI:** 10.1186/s13012-016-0394-6

**Published:** 2016-03-11

**Authors:** Pauline Anrys, Goedele Strauven, Benoit Boland, Olivia Dalleur, Anja Declercq, Jean-Marie Degryse, Jan De Lepeleire, Séverine Henrard, Valérie Lacour, Steven Simoens, Niko Speybroeck, Kris Vanhaecht, Anne Spinewine, Veerle Foulon

**Affiliations:** 1 Louvain Drug Research Institute, Clinical Pharmacy Research Group, Université catholique de Louvain, Brussels, Belgium; 2 Department of Pharmaceutical and Pharmacological Sciences, KU Leuven, Leuven, Belgium; 3 Cliniques universitaires Saint-Luc, Geriatric Medicine, Université catholique de Louvain, Brussels, Belgium; 4 Institute of Health and Society, Université catholique de Louvain, Brussels, Belgium; 5 Cliniques universitaires Saint-Luc, Pharmacy Department, Université catholique de Louvain, Brussels, Belgium; 6 LUCAS–Centre for Care Research and Consultancy and Faculty of Social Sciences, Centre for Sociological Research, KU Leuven, Leuven, Belgium; 7 Department of Public Health and Primary Care, KU Leuven, Leuven, Belgium; 8 Department Public Health and Primary Care, ACHG, KU Leuven, Leuven, Belgium; 9 Faculté de pharmacie et des sciences biomédicales, Université catholique de Louvain, Brussels, Belgium; 10 Institute for Healthcare Policy, Department of Public Health, KU Leuven, Leuven, Belgium; 11 CHU UCL Namur, Pharmacy Department, Université catholique de Louvain, Yvoir, Belgium

**Keywords:** Inappropriate prescribing, Residential facilities, Aged, Complex intervention, Patient care team, Interdisciplinary case conferences, Drug-related problems, Medication review, Medication use

## Abstract

**Background:**

Ageing has become a worldwide reality and presents new challenges for the health-care system. Research has shown that potentially inappropriate prescribing, both potentially inappropriate medications and potentially prescribing omissions, is highly prevalent in older people, especially in the nursing home setting. The presence of potentially inappropriate medications/potentially prescribing omissions is associated with adverse drug events, hospitalisations, mortality and health-care costs. The Collaborative approach to Optimise MEdication use for Older people in Nursing homes (COME-ON) study aims to evaluate the effect of a complex, multifaceted intervention, including interdisciplinary case conferences, on the appropriateness of prescribing of medicines for older people in Belgian nursing homes.

**Methods/design:**

A multicentre cluster-controlled trial is set up in 63 Belgian nursing homes (30 intervention; 33 control). In each of these nursing homes, 35 residents (≥65 years) are selected for participation. The complex, multifaceted intervention comprises (i) health-care professional education and training, (ii) local concertation (discussion on the appropriate use of at least one medication class at the level of the nursing home) and (iii) repeated interdisciplinary case conferences between general practitioner, nurse and pharmacist to perform medication review for each included nursing home resident. The control group works as usual. The study period lasts 15 months. The primary outcome measures relate to the appropriateness of prescribing and are defined as (1) among residents who had at least one potentially inappropriate medication/potentially prescribing omission at baseline, the proportion of them for whom there is a decrease of at least one of these potentially inappropriate medications/potentially prescribing omissions at the end of study, and (2) among all residents, the proportion of them for whom at least one new potentially inappropriate medication/potentially prescribing omission is present at the end of the study, compared to baseline. The secondary outcome measures include individual components of appropriateness of prescribing, medication use, outcomes of the case conferences, clinical outcomes and costs. A process evaluation (focusing on implementation, causal mechanisms and contextual factors) will be conducted alongside the study.

**Discussion:**

The COME-ON study will contribute to a growing body of knowledge concerning the effect of complex interventions on the use of medicines in the nursing home setting, and on factors influencing their effect. The results will inform policymakers on strategies to implement in the near future.

**Trial registration:**

Current Controlled Trials ISRCTN66138978

## Background

Population ageing presents new challenges for the health-care system [1]; optimising medicines’ prescribing is a major aspect in this regard [2]. Many studies across different health-care settings have revealed a high prevalence of potentially inappropriate prescribing in people aged 65 and older [3–5]. The term ‘inappropriate prescribing’ encompasses three categories: overprescribing, referring to the prescription of a drug without a valid indication; misprescribing, referring to incorrectly prescribing a drug for a valid indication; and underprescribing, referring to the failure to prescribe indicated drugs [2]. The presence of inappropriate prescribing has been associated with adverse drug events (ADEs) [6], hospitalisations [6–10] and mortality [7].

Over the last years, explicit (criterion-based) and implicit (judgement-based) tools have been developed to identify inappropriate prescribing [2]. A European explicit tool that is currently widely used allows the identification of ‘potentially inappropriate medications’ or PIMs (i.e. events of overprescribing and misprescribing) on the basis of the Screening Tool of Older People’s Prescriptions (STOPP) criteria and ‘potentially prescribing omissions’ or PPOs (i.e. events of underprescribing) on the basis of the Screening Tool to Alert to Right Treatment (START) criteria [11]. A second widely used explicit tool is the American Beers list [12]. Beers criteria allow identification of PIMs but not PPOs [12].

The prevalence of inappropriate prescribing is particularly high in nursing homes (NHs). Over the past 10 years, several studies have been conducted to identify interventions that can increase appropriate prescribing in this specific setting. Different types of interventions have been tested: educational approaches; pharmacists’ medication reviews; multidisciplinary team approaches and the use of computerised decision support systems (CDSS) [13–18]. These interventions have been tested separately or in combination. Narrative and systematic reviews on this topic showed mixed results [13–18]. This lack of robust conclusions can partly be explained by variability in the intervention, in the outcomes measured [14, 17] and in the quality of the studies [18]. Authors’ conclusions in the different reviews are quite similar: some interventions led to the identification and resolution of drug-related problems (DRPs) [17], but there is no evidence of an effect of the interventions on resident-related outcomes, namely ADEs, hospital admissions and mortality [14, 17, 18] and few or no study assessed quality of life [17]. Authors suggested that a comprehensive approach, including more than one method to improve the quality of prescribing, is likely to be required [13, 15, 18, 19]. As health-care professionals (HCPs) receive little training in pharmacotherapy in older people during their basic education, Forsetlund et al. insisted that any intervention should imply some kind of education [18]. Currently, the right combination of interventions that can improve the quality of prescribing in the nursing home setting is not clear. Moreover, as pointed out by Sanford et al., there is a heterogeneity in the nursing home characteristics between countries (number of beds, funding, nursing home residents (NHRs), trained or untrained staff, visit of physicians,…); hence, generalisation across countries is limited [20].

As most of the interventions tested were complex and multifaceted, some authors pointed out the lack of studies that explored this ‘black box’ effect through a process evaluation to understand the contribution and impact of each component [17, 18].

In Belgium, 8.4 % of the 65+ [21] and 42 % of the 85+ live in a NH [22]. Each Belgian NH must appoint a coordinating physician, who is, together with the head nurse, responsible for the therapeutic policy of the NH. Medication-related tasks include organising medication management in collaboration with pharmacists, developing and updating the medication formulary, organising training, etc. Each NHR can choose his/her general practitioner (GP); consequently, the number of visiting GPs is unrestricted. Furthermore, the GP has a total freedom of diagnostic and therapeutic strategies, although he is stimulated to follow the therapeutic policy of the NH. The delivery of medication is performed by pharmacies, either hospital pharmacies or community pharmacies. The NH is in charge of the choice of the delivering pharmacy.

A study conducted in Belgian NHs in 2006 found that the median number of chronic medications per patient was 8 (5–10) [22]. Fifty-three percent of residents received 5 to 9 chronic medications daily and 20.8 % had 10 to 14 chronic medications [22]. A particularly high consumption was observed for benzodiazepines and antipsychotics (68 %), laxatives (50 %) and antidepressants (46 %) [22]. In another Belgian study, more than 50 % of NHRs had at least one drug that could be stopped or modified according to the STOPP criteria, while for 30 % of the residents at least one drug was missing, based on the START criteria [23].

While policy approaches to improve the quality of drug prescribing in the nursing home setting have been adopted in several developed countries such as the USA, UK, Australia and New Zealand [24], no similar action has yet been set up in Belgium.

Given the high consumption of medications, the high prevalence of inappropriate use of medications, and the lack of structured interdisciplinary approaches in Belgian NHs, the Belgian National Institute for Health and Disability Insurance (NIHDI) launched a national call in 2013. The objective was to perform a national quality improvement pilot project, aiming at evaluating the effect of interdisciplinary collaboration on the appropriateness of use of medications by NHRs and developing recommendations to policymakers. This pilot project is the Collaborative approach to Optimise MEdication use for Older people in Nursing homes (COME-ON) study described hereafter.

### Objectives

The primary objective of the COME-ON study is to evaluate the effectiveness of a complex, multifaceted intervention on the appropriateness of prescribing for older people in Belgian NHs. General practitioners, coordinating physicians, nurses and pharmacists are involved in the COME-ON study. The intervention is built up from a number of components, mainly training, local concertation and interdisciplinary medication reviews during case conferences. These components may act both independently and inter-dependently. The intervention can therefore be considered as a complex intervention [25].

The secondary objectives are to evaluate the effect of the intervention on medication use, on resident outcomes in terms of hospital admissions and visits to the emergency department, and on costs. In addition, a process evaluation will be conducted to examine the extent to which the intervention is implemented as planned and to explore participants’ experiences regarding reasons of success or failure, barriers and facilitators.

The study development was informed by the Medical Research Council (MRC) guidance for developing and evaluating complex interventions [25]. The COME-ON study is built on (a) learning from previous research [15, 17, 18, 26], (b) focus groups with HCPs working in Belgian NHs to identify their needs with regard to optimization of medication use, and (c) a pilot study performed in four NHs spread in the three regions of Belgium. During this pilot study, the recruitment approach, the adequacy of research instruments and the feasibility and acceptability of interdisciplinary medication review were assessed.

## Methods/design

### Trial design

The COME-ON study is a multicentre, cluster, parallel-group controlled trial. Each NH represents one cluster. As the intervention is mainly provided at the level of HCPs, a cluster design was chosen to prevent contamination bias. The study design was developed in accordance with the Consolidated Standards of Reporting Trials (CONSORT) statement extension to cluster RCTs [27].

### Participating nursing homes (clusters)

**Fig. 1:**
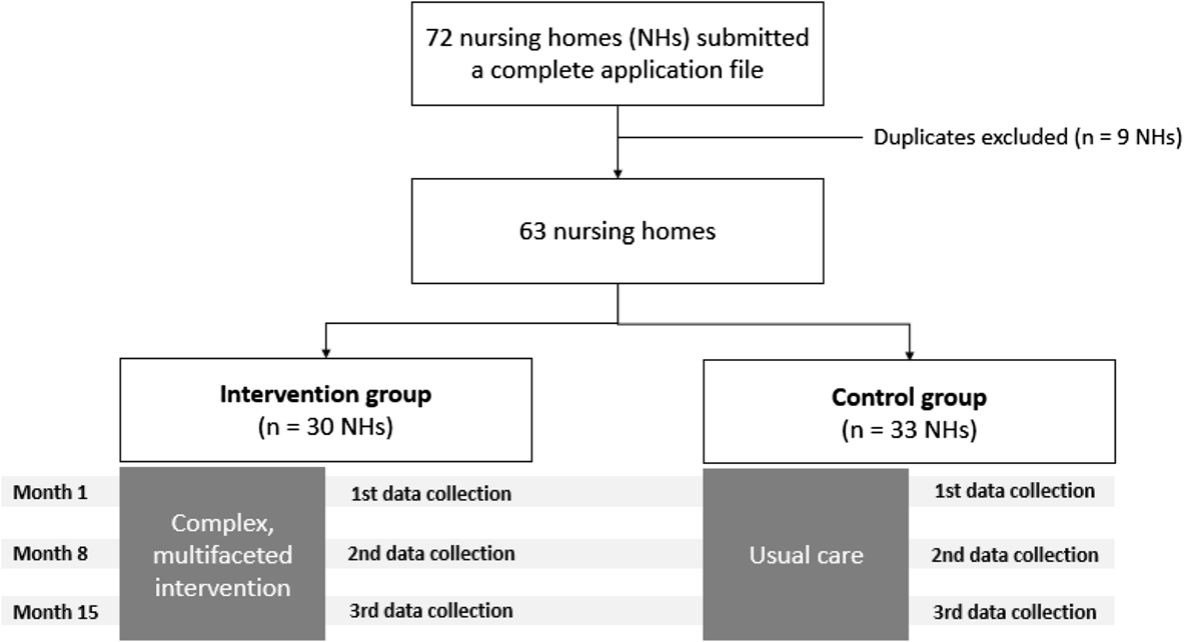
COME-ON study flowchart

In the summer of 2013, a national call addressed to all Belgian NHs was launched by NIHDI. Nursing homes with at least 35 residents were eligible to participate. For each NH willing to participate, the management board, coordinating physician and delivering pharmacist were asked to complete and sign an application file with descriptive data relative to the NH, to the residents and to the local medication management process. Each coordinating physician, pharmacist and management board could not be involved in more than one application (to maximise variability in the sample and to prevent contamination bias). In total, 72 NHs applied. After exclusion of duplicates (i.e. two or more applications with the same pharmacist, the same coordinating physician and/or the same management board), a final list of 63 NHs was obtained (Fig. 1).

### Allocation

Nursing homes were stratified by (a) province/region, (b) experience with multidisciplinary case conferences (as reported in the application file) and (c) type of medication delivery (by a hospital pharmacy or by one or more community pharmacies). Factors (b) and (c) were considered to be possibly significant covariates. Geographical location (factor a) was taken into account because the funding body (NIHDI), requested that (i) at least one NH from each province/region was given the opportunity to implement the intervention and (ii) the number of NHs allocated to each group was balanced per province/region.

In 4 of the 10 Belgian regions/provinces, only one NH applied. These 4 NHs were therefore immediately assigned to the intervention group. The characteristics of the other 59 NHs with regard to location (province/region), experience with case conferences and type of delivering pharmacy, were entered into Statistical Package for the Social Sciences (SPSS; version 22). This programme generated a series of blocks for each stratum and allocated NHs to control or intervention group randomly within each block. Allocation was performed by an independent researcher blinded to the identification of the NHs and not involved in the recruitment of NHs or residents. Thirty NHs were allocated to the intervention group (as stipulated by NIHDI) and 33 to the control group. Because of the nature of the intervention, it was not possible to blind NHs or HCPs to the intervention.

### Nursing homes residents

Residents were considered eligible if they were aged 65 years or older and were under the care of a participating GP. Before the study started, the coordinating physician contacted all GPs who had at least one resident in the NH to ask for their willingness to participate in the study. Residents receiving palliative care (according to the GP’s evaluation) and residents in subacute care/rehabilitation were excluded.

### Recruitment

In order to have a sufficient (to improve generalisability) but reasonable (for convenience reasons) number of GPs involved, all participating GPs caring for at least three eligible residents were identified. If possible, a maximum of 12 GPs per NH was aimed for. The objective was to include 35 residents per NH. One member of the research team performed resident selection assisted by the head nurse, the coordinating physician or a representative of the management board. An algorithm was developed (available upon request) to support NHR recruitment. If there were more than 35 eligible residents, a random selection was made using a statistical software (Statistical Package for the Social Sciences (SPSS) version 22). Written informed consent was asked by physicians (GPs or coordinating physicians) or nurses to each resident or to a resident’s representative if the resident was not mentally competent (e.g. in case of severe dementia). Due to practical reasons, resident inclusion could not be performed before group allocation.

### Intervention

**Fig. 2:**
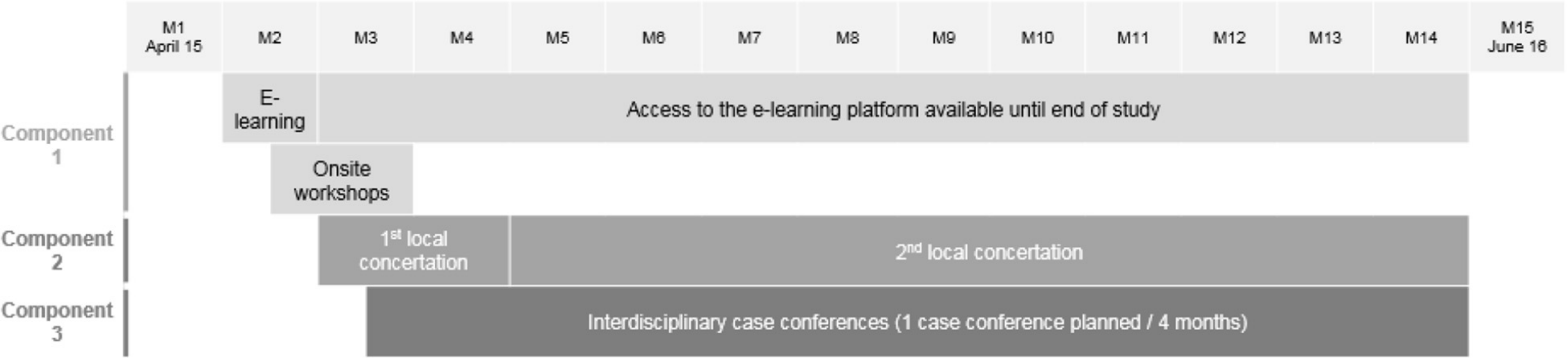
Timeline of COME-ON intervention

Based on the evidence that the most successful interventions tend to be interdisciplinary and include more than one method [15, 17, 18, 26], a multifaceted strategy was chosen. The key element of this complex intervention is the structured and repeated interdisciplinary resident’s medication review (referred to hereafter as ‘interdisciplinary case conferences’) supported by training and local concertation. Each component of the intervention is described hereafter and in Table 1. The timeline is provided in Fig. 2. The main components of the complex intervention have been tested during the pilot phase and were refined afterwards.

**Table 1:** Intervention components

Intervention component	Description	Participants involved	Moment and frequency	Incentives
Training—e-learning	Module 1: Pharmacotherapy in older people; potentially inappropriate medications and tools used to measure it Module 2: What is a medication review? How can each health care professional contribute? Module 3: How to perform an interdisciplinary medication review? Module 4: Teamwork	Physicians Nurses Pharmacists	Available during whole study period	Accreditation for GPs and pharmacists; certificate of attendance for nurses
Training—onsite workshops	Specific training for pharmacists, provided by the research team: ▪ How to prepare a medication review? How to make a suggestion? Where to find relevant information about medications?	Pharmacists	At month 2	Accreditation for pharmacists
	Training for all HCPs, provided by the research team: ▪ Problem-based learning using clinical vignettes: How to conduct an interdisciplinary medication review? What is the contribution of each HCP? How to classify DRPs?	Physicians Nurses Pharmacists	At month 2	Accreditation for GPs and pharmacists; certificate of attendance for nurses
	Specific training for nurses, provided by the coordinating physician and/or pharmacist: ▪ Detection of adverse drug events by nurses ▪ Drug administration	Nurses	Twice or more during the study period	Certificate of attendance
Local concertation	Discussion about the use of two classes of medications (antidepressants and lipid-lowering drugs) at the level of the nursing home	Physicians Nurses Pharmacists	Twice during the study (first at month 3 or 4)	Remuneration for all HCPs involved in the COME-ON study
Interdisciplinary case conferences	Face-to-face medication reviews for each included NHR; duration approximately 20 min	Physicians Nurses Pharmacists	Once per 4 months	Remuneration for all HCPs

*DRP*(s) drug-related problem(s), *GP*(s) general practitioner(s), *HCPs* health-care professionals, *NHR* nursing home resident

### Component 1: education and training

A blended learning programme, combining e-learning with face-to-face workshops, has been developed. Training needs and desired formats were discussed during focus groups with HCPs. The structure of the e-learning platform, the format and content of the blended learning programme were developed in collaboration with a team of HCPs and experts in e-learning and geriatric pharmacotherapy.

The e-learning consists of four modules on the following topics: drugs and ageing; (in)appropriate prescribing; medication review; and team work. Each module takes approximately 60 min to complete and is built up from a variety of learning formats including narrative powerpoints, videos, serious games, assignments, summary tools on specific topics and tests. All participating HCPs from NHs allocated to the intervention group have access to the e-learning platform that is available during the whole study duration.

Educational material developed by the research team is also available through the e-learning platform. It includes a medication review flowchart, a shortlist of STOPP/START criteria, and summary sheets on different topics (e.g. renal function, anticholinergic drugs,…).

Furthermore, the research team moderates two types of face-to-face interactive workshops (i.e. onsite training), 2 h each, one specific for pharmacists and one with all participating HCPs of one or more NHs. The aim is to apply the theoretical concepts, addressed during the e-learning, to clinical cases, and to get familiar with research instruments (DRP classification; web application). Specific onsite training for nurses can be given by the coordinating physician and/or the pharmacist. To encourage this type of training, preparatory material on administration of medications and on detection of ADEs is provided by the research team.

As an incentive, the e-learning modules and onsite workshops are accredited for GPs as well as for pharmacists. As there is currently no similar accreditation programme for nurses, they receive a certificate of attendance.

### Component 2: local concertation

At the level of each participating NH, physicians (GPs and coordinating physician), pharmacists and nurses are asked to participate in two ‘local concertation’ meetings. The objectives of this component are (a) to reach consensus on the appropriate use of one specific class of medication within each NH, with the intent that this work could then be used during the interdisciplinary case conferences and (b) to initiate teamwork and communication between HCPs of the same NH. The overall output is expected to be (i) a ‘vision’ or a ‘management plan’ for the treatment of certain condition(s), (ii) a list of (in)valid indications for the use of the discussed medications; (iii) a list of molecules to be preferred or avoided, and underlying reasons and (iv) modalities of the use of the preferred drugs (e.g. dosage, duration, follow-up,…). Two medication classes—antidepressants and lipid-lowering drugs—were selected by the research team. Material to support the preparation of the meetings and the discussion is provided by the research team.

Each meeting is expected to last approximately 2 h. The coordinating physician, the pharmacist and the head nurse are invited to take the lead in preparing, organising and implementing the meetings (i.e. invitations, content, discussion, guidelines, overview of figures and numbers of NHRs taking these medications, summary, report). Depending on the opportunities and willingness, additional HCPs can be invited to participate (e.g. geriatrician, psychiatrist, physiotherapist, occupational therapist). The first meeting has to be held at the beginning of the study, ideally before the first interdisciplinary case conferences. A second meeting takes place several months later. The objective of the latter is to review the implementation of the consensus reached after the first meeting, to re-discuss or amend this consensus if necessary, and/or to start the discussion about the second medication class. The HCPs involved in the study are paid for their attendance at the meetings.

### Component 3: interdisciplinary case conferences

Face-to-face medication reviews have to be conducted for each NHR included in the study, by an interdisciplinary team consisting of three HCPs: the GP, the pharmacist, and a nurse (head nurse, or other nurse involved in the care of the resident). The medication review focuses on the appropriate and cost-effective use of all medications taken by the resident. During the discussion, the team determines whether drugs must be additionally prescribed, tapered, discontinued, dose-adjusted, or replaced, and whether other actions are needed. They are also requested to prioritise and time schedule the treatment modifications to be made. Drug-related problems and interventions must be recorded using a DRP classification tool adapted from Basger et al. [28] and from the PCNE classification V6.2 [29].

The interdisciplinary case conferences are facilitated by the use of a web application. This web application was primarily developed for allowing electronic data collection (clinical, medical, economic and medication data). It also enables (i) sharing data about NHRs between HCPs in the intervention group, allowing preparation of the medication review by each HCP and (ii) generating a standardised report of every case conference, including details on DRPs and interventions, with the possibility to re-discuss or amend these interventions at the next meeting.

A total of three case conferences per resident over a 12-month period are aimed for. Each case conference is estimated to last about 20 min. For residents with a hospital admission during the study period, HCPs are encouraged to perform an additional medication review in the fortnight after hospital discharge. For residents entering end-of-life care, an additional medication review can be conducted with a focus on stopping unnecessary medications and optimising comfort.

NHRs do not participate in the case conferences. However, the nurse and the GP are supposed to represent the interest of the residents by sharing information on the perception and preferences of the NHRs regarding their current medication regimen. NHRs and/or their family will be given the opportunity to receive feedback from the nurse or the GP on the issues discussed during the case conferences, and to get information on the treatment and the proposed changes. HCPs can agree to postpone the implementation of certain interventions until discussion with and agreement of the NHR and/or family. The HCPs are paid for each interdisciplinary case conference.

### Control group

Nursing homes allocated to the control group receive no intervention and continue delivering usual care. After completion of the last data collection, control NHs will have access to the e-learning platform and will have the option of attending a symposium that presents a summary of key messages relative to the effect of the intervention. They will also receive feedback on their own results relative to appropriateness of prescribing.

### Data collection

Data will be collected at three points in time: at month 1 (baseline), month 8 and month 15 (end of study). Data collection is performed by the HCPs involved in the care of each resident through a web application. Administrative and clinical data (e.g. age, functional status) are collected by the nurse, comorbidities (past and current medical history) and laboratory values by the GP, and medication data by the pharmacist. In the middle (month 8) and at the end of the study (month 15), data on health-care use (hospital admissions, emergency visits, GP or specialist visits) will also be collected by the nurse and the GP.

Characteristics of NHs (e.g. number of beds, location) and administrative data about participating HCPs are collected at the beginning of the study (month 1) from the coordinating physician and respective HCPs. HCPs will be paid for data collection.

In the intervention group, HCPs are also requested to record data on each case conference (participants, time for preparation, duration, DRPs identified, interventions,…)

Access to the web application is secured and requires both the national electronic identity card and the entry on a list of registered HCPs. HCPs have only access to data collection files of NHRs for which they are responsible. Data export from the web application to the research database will be performed with the intervention of a trusted third party (TTP), who is responsible for data coding and small cell analysis. NHRs and HCPs will be known to the research team by study ID number only. The whole process was approved by the Privacy Commission (nb 14/095, 21/10/2014).

For qualitative data (see ‘Process evaluation’ section), all interviews and focus groups will be audio recorded and transcribed verbatim, and the transcripts will be pseudo-anonymised.

### Outcomes

#### Primary outcome

The primary outcome measures relate to the appropriateness of prescribing at the resident level and are defined as (1) among residents who had at least one PIM/PPO at baseline, the proportion of them for whom there is a decrease of at least one of these PIM/PPO at the end of study and (2) among all residents, the proportion of them for whom at least one new PIM/PPO is present at the end of the study as compared to baseline.

PIMs/PPOs will be identified from a pre-defined list of explicit criteria that includes STOPP/START (version 2) [11] and Beers (2015) criteria [12]. To automatically identify PIMs and PPOs from the research database, the research team has developed and validated with GPs, geriatricians and pharmacists an algorithm for detecting STOPP/START criteria (version 2) and Beers criteria (2015).

### Secondary outcome measures

The secondary outcome measures include individual components of appropriateness of prescribing, medication use, the outcomes of the case conferences, clinical outcomes and costs. An overview of secondary outcome measures is provided in Table 2.

**Table 2:** Secondary outcome measures and process evaluation

	Measure
Secondary outcome measures	
Appropriateness of prescribing, individual components	-Prevalence of PIM and PPO per criterion
Medication use	-Number of medications per NHR -Classes of medications used (ATC level 2 and 3)
Clinical outcomes	-Death -Hospital admissions -Visits to an emergency department -Visits to the GP and to specialist physicians
Outcomes of case conferences	-Type of identified DRPs -Type of planned interventions -Mean/median number of DRPs identified per NHR -Proportion of interventions implemented at the next case conference
Cost	-Cost of medication -Cost of healthcare use (hospital admissions, visits to the emergency department, GP visits, consultation with specialists) -Cost of intervention
Process evaluation	
Training	-Dose (number of module per profession,…) -Reach (rate of participation,…) -Experience with intervention (satisfaction survey)
Local concertation	-Fidelity (percentage of NHs that organised 2 local concertations,…) -Dose (number of local concertations per NH) -Reach (number of participating GPs who attended local concertation,…) -Experience with intervention (semi-structured interviews and focus groups)
Interdisciplinary case conferences	-Fidelity (percentage of NHRs for which 3 interdisciplinary case conferences were conducted,…) -Dose (median number of case conferences per NHR,…) -Reach (who attended case conferences,…) -Experience with intervention (semi-structured interviews and focus groups)
Context of the NH	-Characteristics of NHs and HCPs -Context in which the intervention is being implemented (semi-structured interviews)

*PIM* potentially inappropriate medication, *PPO* potentially prescribing omission, *NHR*(s) nursing home resident(s), *GP* general practitioner, *NH*(s) nursing home(s), *DRP*(s) drug-related problem(s), *HCPs* health-care professionals

#### Process evaluation

MRC guidance for complex intervention studies recommends that process evaluations must be conducted within the trial to assess the implementation of the intervention, to clarify causal mechanisms and to identify contextual factors associated with variation in outcomes [30]. These three aspects (i.e. implementation, causal mechanisms and contextual factors) will be evaluated during the COME-ON study (see Table 2). For the implementation evaluation, we will evaluate (1) fidelity, which is the quality of the implementation, and whether the intervention was implemented as intended in the protocol; 2) dose, which is the number of components of the intervention that was delivered; and 3) reach, which is the extent to which the target audience came into contact with the intervention.

A combination of quantitative and qualitative methods (i.e. mixed-method approach) will be used. The qualitative process evaluation will be conducted through interviews or focus groups with a purposive sample of HCPs to explore their views on the acceptability, effectiveness, barriers and facilitators of the intervention. Interview guides will be developed and piloted.

### Sample size

Estimations of sample size calculations were performed for the two primary outcomes.

1. First, we estimated that among residents who had at least one PIM/PPO at baseline, there would be a decrease of at least one of these PIM/PPO at the end of the study for 20 % of NHRs in the control group and 40 % in the intervention group. We considered a power of 80 % and a statistical significance of 5 %. To take into account, the cluster nature of the study, we inflated the standard sample size estimates by a factor, i.e. the design effect = 1 + *ρ*(*m* − 1), where *m* is the average cluster size and *ρ* is the estimated intracluster correlation coefficient (ICC) [31]. As Belgian data on the ICC for the primary outcome measure were not available, we made estimations for two extreme values, i.e. 0.05 and 0.5. In fact, based on what has been found in the other primary care research setting, the ICC can be reasonably estimated between 0.05 and 0.5. With an average cluster size of 30 NHRs and an estimated percentage of dropouts of 22 %, a total of number of 502 and 3175 NHRs will be required for ICC values of 0.05 and 0.5, respectively.
2. Second, we estimated that among all residents, there would be at least one new PIM/PPO present at the end of the study as compared to baseline for 20 % of NHRs in the control group and 10 % in the intervention group. Similarly to the estimation for the first outcome, we considered two extreme values of ICC, i.e. 0.05 and 0.5. With an average cluster size of 30 NHRs and an estimated percentage of dropouts of 22 %, a total of number of 1223 and 7738 NHRs will be required.

As mentioned above, the funding body (NHIDI) determined a priori the number of NHs and NHRs for the COME-ON study. Based on the previous cluster studies, we can expect an ICC closer to 0.05 than 0.5; consequently, a total of approximately 1890 NHRs might be enough to detect a statistically significant difference.

### Data analysis

#### Quantitative analysis

Data from the trial will be analysed and reported in accordance with the CONSORT criteria. The baseline characteristics will be reported as mean (± standard deviation) or median (and P25; P75) values for continuous variables and as counts (percent) for categorical variables. Descriptive statistics will be used to evaluate differences between intervention and control group on baseline characteristics at the resident, physician and nursing home level. Continuous variables will be compared using Student *T* test or Wilcoxon rank-sum test for clustered data, as appropriate. Categorical variables will be compared using adjusted chi-square test for clustered data.

To assess the impact of the intervention on the primary outcomes, logistic regression will take into account the complex design issues of the study (clustering, stratification). A multivariable model will be built to adjust the analysis for potential confounders, i.e. the number of PIMs/PPOs at baseline per resident and other baseline characteristics of residents, GPs and NHs. The analysis will be conducted using the intent-to-treat analysis, i.e. all residents will be analysed as randomised. A per protocol analysis will also be performed as a secondary analysis.

The impact of the complex intervention on the secondary outcome measures will be assessed using descriptive data and in some cases, using appropriate statistical evaluation.

Data analysis will be performed using R software (Free Software Foundation, Inc., Boston, MA, USA). A *p* value <0.05 will be considered statistically significant.

### Cost analysis

For the cost analysis, the mean treatment cost over the study period (from baseline till the end of the study) will be compared between the residents in the control and in the intervention group. Treatment costs will be compared using the independent samples *t* test for variables having a normal distribution or the Mann-Whitney *U* test for variables not having a normal distribution. These costs will be calculated in 2015 and 2016 values from the perspective of the health-care payer (NIHDI contribution plus patient co-payment, if applicable). Costs will include the costs of the intervention, the costs of medication and the costs of other health-care use (GP visits, consultations with specialised physicians, visits to emergency departments and hospitalisations). With respect to the latter, the duration of hospitalisations will be multiplied by the per diem hospitalisation prices to generate a proxy estimate of hospitalisation costs (this excludes hospital costs for medication, medical imaging and clinical biology, for which no data will be available) [32].

### Qualitative analysis

All interviews and/or focus groups will be audio-recorded and transcribed verbatim. A thematic framework analysis will be conducted, and facilitated through the use of NVivo 10.

### Ethical approval

The study has been approved by the Ethics Committee of UZ Leuven on November 12, 2014 (reference number s57145, ML11035). Furthermore, this study has also been approved by the Belgian Privacy Commission on October 21, 2014 (Beraadslaging nr. 14/095, reference: SCSZ/14/084/174). All included NHRs or residents’ representatives provided their written informed consent before the beginning of the study.

### Study registration

This study has been registered at http://www.isrctn.com/(trial registration number: ISRCTN66138978)

### Trial status

Recruitment of NHRs started in January 2015 and was followed by the baseline data collection in April 2015. The complex intervention was implemented in NHs from May 2015 onwards, and currently, the second data collection is ongoing.

## Discussion

Articles that have been published in recent years have highlighted the need to find strategies to optimise the quality of prescribing in the older population. The COME-ON study assesses the effectiveness of a complex, multifaceted intervention on the appropriateness of prescribing in the nursing home setting.

To our knowledge, this is the first Belgian cluster controlled trial and the largest European trial that assesses a collaborative approach to optimise medication use in the nursing home setting.

As recommended in narrative and systematic reviews on this topic, the strength of this study is the use of a multifaceted intervention, including different aspects and comprising an educational part. Moreover, the intervention is supported by a web-application that allows sharing information between all HCPs involved. The intervention is also conducted in real-life context; in fact, the intervention relies on HCPs actually working in the nursing home setting. Thus, if the intervention proves to be effective, it could be implemented more easily on a wider scale. In the assessment of a complex intervention, the scope of evaluation should be broader than effectiveness; in fact, policy makers need information on what works, how and why, for interpreting the findings and generalising the intervention on a larger scale [30]. This is why a process evaluation is conducted alongside the study.

There are also potential limitations to this study. First, the included NHs and participating GPs gave their agreement to participate in the study; thus, they are perhaps more concerned about the issue of drug prescribing for older people. Second, NHRs could only be included if their treating GP was willing to participate. Third, this study uses intermediate and process outcome measures instead of final clinical endpoints.

In conclusion, the COME-ON study will contribute to a growing body of knowledge concerning the effect of complex interventions on the use of medicines in the NH setting, and on factors influencing their effect. The results will inform policymakers on strategies to implement in the near future.

## Abbreviations

ADEs: adverse drug events
DRP(s): drug-related problem(s)
GP(s): general practitioner(s)
HCPs: health-care professionals
ICC: intracluster correlation coefficient
NH(s): nursing home(s)
NHR(s): nursing home resident(s)
NIHDI: National Institute for Health and Disability Insurance
PIM(s): potentially inappropriate medication(s)
PPO(s): potentially prescribing omission(s)
